# Therapeutic suppression of *Helicobacter pylori* infection in C57BL/6J mice using specific polyclonal antibodies targeting BabA and FrpB4

**DOI:** 10.3389/fmicb.2026.1867770

**Published:** 2026-07-31

**Authors:** Lu Zhou, Yusen Wei, Jiapei Hu, Yongxian Xue, Yutong Nie, Zhongli Shi, Dailun Hu

**Affiliations:** 1 Graduate School, Hebei Medical University, Shijiazhuang, China; 2 Department of Oncology, Hebei General Hospital, Shijiazhuang, China; 3 Institute of Basic Medicine, Hebei Medical University, Shijiazhuang, China; 4 Hebei Key Laboratory of Brain Science and Psychiatric-Psychologic Disease, The First Hospital of Hebei Medical University, Shijiazhuang, China; 5 Department of Pathogenic Biology, Hebei Medical University, Shijiazhuang, China

**Keywords:** antiserum, BabA, FrpB4, *Helicobacter pylori*, immunotherapy

## Abstract

**Background:**

Current eradication therapies for *Helicobacter pylori (H. pylori*) are increasingly compromised by the emergence of antibiotic resistance and adverse side effects. Exploration of non-antibiotic, targeted therapeutic alternatives is essential for effective *H. pylori* management.

**Aim:**

To evaluate the therapeutic efficacy of a rationally designed specific antiserum targeting a BabA_189−244_-FrpB4_380−395_ fusion peptide against *H. pylori* infection.

**Methods:**

Using immunoinformatics, the Le^b^ antigen binding domain of BabA (residues 189–244) and the immunogenic regions of FrpB4 (residues 380–395) were selected and synthesized as a fusion peptide. This engineered peptide was used to immunize rabbits to generate specific polyclonal antiserum. The direct antimicrobial effect was evaluated *via* an *in vitro* disk diffusion assay, and its impact on *H. pylori* adhesion to AGS (human gastric adenocarcinoma cell line) was assessed following bacterial pre-incubation with the antiserum. *In vivo*, C57BL/6J mice were inoculated intragastrically with *H. pylori* (1 × 10^9^ CFU). After confirming bacterial colonization in the gastric mucosa, the infected mice (n=6 per group) received a daily oral administration of the antiserum (0.2 mL) for 2 weeks. Therapeutic efficacy was quantified *via* gastric bacterial load counting, urease activity measurement, and histological evaluation.

**Results:**

Immunization with the BabA_189−244_-FrpB4_380−395_ fusion peptide successfully elicited high-titer specific antibodies. *In vitro*, the antiserum significantly restricted *H. pylori* growth and reduced bacterial adherence to AGS cells (*P* < 0.05). *In vivo*, 2 weeks of oral antiserum administration markedly reduced the gastric bacterial burden, suppressed mucosal urease activity (*P* < 0.05), and attenuated histological colonization compared with untreated controls.

**Conclusions:**

The specific antiserum targeting the BabA-FrpB4 fusion peptide exhibits significant antimicrobial and anti-adhesive activities against *H. pylori*, effectively restricting gastric colonization *in vivo*. This immunotherapeutic approach represents a viable non-antibiotic strategy for *H. pylori* intervention.

## 1 Introduction

*Helicobacter pylori* (*H. pylori*), a Gram-negative pathogen, can establish persistent colonization in the human stomach. Affecting nearly half of the world's population, *H. pylori* is a significant global health concern (Li et al., 2023). The infection contributes to various gastrointestinal disorders, including chronic gastritis, peptic ulcer disease, mucosa-associated lymphoid tissue (MALT) lymphoma, and gastric adenocarcinoma, alongside certain extragastric manifestations (Gravina et al., 2018; Reshetnyak et al., 2021; Chen et al., 2019). Despite its high prevalence and pathogenic potential, achieving sustainable eradication faces increasing clinical challenges.

Current first-line antimicrobial therapies for *H. pylori* rely on triple or quadruple antibiotic regimens, often combined with proton pump inhibitors (Fallone et al., 2019; Malfertheiner et al., 2022). However, the clinical efficacy of these conventional treatments is declining due to rising antimicrobial resistance, adverse effects, and poor patient adherence, leading to elevated treatment failure rates (Fallone et al., 2019; Nguyen et al., 2019; Yu et al., 2024). These limitations underscore the need for alternative, non-antibiotic interventions, including targeted approaches utilizing specific antibodies for pathogen clearance.

Passive immunotherapy utilizing specific antibodies offers a potential alternative for localized infection control. Early strategies employing polyclonal antibodies derived from bovine or avian models demonstrated preliminary prophylactic and therapeutic effects. However, they faced translational barriers due to broad antigen targeting, batch-to-batch variability, and potential cross-reactivity (Marnila et al., 2003; Shin et al., 2002; Hu et al., 2015; Rokka et al., 2008). To address these issues, synthetic peptide antigens have emerged as a targeted approach. Peptides can be engineered to mimic conserved, surface-exposed domains of bacterial targets, aiming to generate specific neutralizing antibodies while minimizing off-target binding. Supported by structural biology and immunoinformatics, computational pipelines now facilitate the prediction and selection of immunogenic epitopes (Rappuoli et al., 2016; Serruto et al., 2012; Meza et al., 2017; Zhang et al., 2022; Keshri et al., 2023).

The selection of appropriate surface targets is a critical step in effective antibody design. Adherence of *H. pylori* is a multifactorial process mediated by various outer membrane proteins (Keilberg and Ottemann, 2016; Xu et al., 2020; Oleastro and Ménard, 2013). BabA serves as a primary candidate target because its atomic structure and receptor-binding domains have been resolved, facilitating the rational design of antibodies intended to introduce precise steric hindrance (Moonens et al., 2016; Ilver et al., 1998). Furthermore, to counterbalance the functional redundancy and geographic strain variability associated with a single adhesin, targeting a conserved metabolic channel provides a complementary intervention pathway. FrpB4 is a TonB-dependent, iron-regulated outer membrane receptor strictly involved in the uptake of essential trace metals, fulfilling a physiological requirement for bacterial survival and replication in the acidic gastric environment (De Reuse et al., 2013; Schauer et al., 2007; Kumar et al., 2022; Wolfram et al., 2006).

In this study, we employed a bioinformatics-driven strategy to design a fusion polypeptide targeting specific functional domains of BabA and FrpB4. This rationally designed multi-target antigen was utilized to elicit specific antiserum in rabbits. We hypothesized that the specific antibodies would exert steric and metabolic suppressive effects. Specifically, steric blockade of BabA partially hinders host-pathogen adherence, while binding to FrpB4 physically obstructs essential metal ion uptake channels to induce nutritional stress. Through *in vitro* assays and an *in vivo* C57BL/6J mouse infection model, we evaluated the therapeutic efficacy of the orally administered specific antiserum. We aimed to demonstrate its capacity to restrict bacterial growth and disrupt colonization through combined physical and metabolic suppression, providing mechanistic data for non-antibiotic intervention strategies.

## 2 Materials and methods

### 2.1 Antigen design and synthesis

#### 2.1.1 Peptide screening and synthesis

**Scheme 1:**
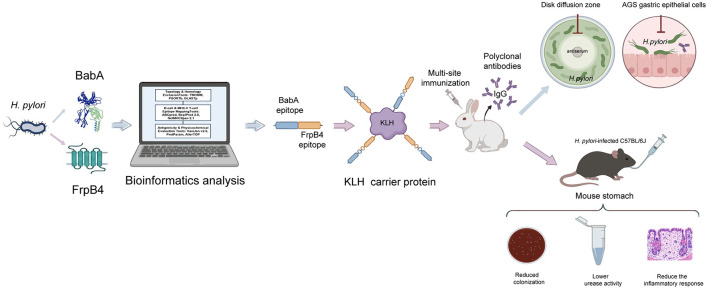
Workflow diagram. Flowchart illustrating the complete experimental workflow from initial antigen design to subsequent *in vitro* and *in vivo* validation.

For the rational design of the fusion antigen (Scheme 1), protein sequences of BabA and FrpB4 from *H. pylori* strain 26695, and representative global strains (*n*≥10; accession numbers provided in Supplementary Tables S1, S2) were retrieved from the UniProt database. To ensure target broad-spectrum recognition, multiple sequence alignments were performed using DNAMAN and CLC Main Workbench v7.8 to define highly conserved functional domains and to generate a consensus sequence. For BabA, structural analyses from previously published data were leveraged to identify sequences within the CL2, DL1, and DL2 domains responsible for Le^b^ antigen binding.

For FrpB4, a comprehensive bioinformatic pipeline was applied to the consensus sequence. Subcellular localization and transmembrane topology were predicted using PSORTb 3.0.3, TMHMM v2.0, and DeepTMHMM to confirm the extracellular orientation of the target domains. To mitigate cross-reactivity and potential autoimmunity risks, sequences exhibiting high homology with the human proteome were excluded *via* BLASTp analysis. Epitope prediction was strictly categorized: linear B-cell epitopes were mapped using ABCpred, SVMTriP, and BepiPred 2.0; MHC-I and MHC-II binding T-cell epitopes were predicted *via* NetMHCpan 4.1 and NetMHCIIpan 2.1, respectively.

Dominant epitopes from both BabA and FrpB4, exhibiting antigenicity scores above the 0.4 threshold (VaxiJen v2.0), were selected for chimera construction. The physicochemical properties of the final fusion construct, including molecular weight and isoelectric point (Compute pI/Mw tool), were calculated using ProtParam. Allergenicity and solubility were evaluated *via* AllerTOP v2.0, AlgPred, and SOLpro servers. Finally, the designed fusion polypeptide was chemically synthesized by Nanjing Peptide Industry Co., Ltd. (Nanjing, China). The molecular mass was verified by mass spectrometry, and the purity was confirmed to be >95% *via* high-performance liquid chromatography (HPLC; Supplementary Figure S1). The synthesized peptide was lyophilized and stored at −80 °C until further use.

### 2.2 Antibody preparation and characterization

#### 2.2.1 Peptide conjugated with keyhole limpet hemocyanin (KLH)

Sulfhydryl-reactive cross-linking chemistry was employed to conjugate the synthetic peptide to KLH (catalog no. H7017, Sigma-Aldrich, St. Louis, MO, USA). Briefly, the synthetic polypeptide was incubated with the carrier protein under optimized buffer conditions to facilitate stable conjugation. The efficiency of the coupling reaction was quantitatively determined using Ellman's reagent (catalog no. 22582, Thermo Fisher Scientific, Waltham, MA, USA) by measuring the consumption of free sulfhydryl groups, thereby ensuring a high conjugation ratio prior to animal immunization.

#### 2.2.2 Immunization and sample collection

Four female Japanese White rabbits (weighing 2.5–3.0 kg; obtained from Liaoning Changsheng Biotechnology Co., Ltd., Liaoning, China) were utilized for the production of polyclonal antiserum. The rabbits were individually housed in a specific pathogen-free facility under standard controlled conditions (temperature 21 ± 2 °C, relative humidity 50 ± 10%, and a 12-h light/dark cycle) with *ad libitum* access to food and water. Animals were closely monitored for any adverse clinical signs throughout the protocol. All animal experiments were approved by the Institutional Animal Care and Use Committee of Hebei Medical University and conducted in strict compliance with institutional and national animal welfare guidelines.

For the primary immunization, the KLH or KLH-conjugated antigen was thoroughly emulsified with an equal volume of Freund's complete adjuvant (Sigma-Aldrich, St. Louis, MO, USA) to yield a final volume of 1 mL. Subsequent booster immunizations were carried out using Freund's incomplete adjuvant (Sigma-Aldrich) at a 1:1 ratio, following the schedule detailed in Table 1. All immunizations were administered *via* multi-site subcutaneous injections along the dorsal region.

**Table 1:** Immunization dose in rabbits.

Immunization round	KLH (μg)	Antigen polypeptide (μg)	Adjuvant (μL)
First immunization	500	250	500 Freund's complete adjuvant
Second immunization	250	125	500 Freund's incomplete adjuvant
Third immunization	250	125	500 Freund's incomplete adjuvant

Pre-immune serum and serial blood samples were collected for monitoring antibody titer dynamics and obtaining terminal antiserum. Serial blood samples were collected from the central ear artery 7 days after immunization. All specimens were allowed to clot at room temperature for 30 min, centrifuged at 1,000 × g for 5 min at 4 °C to separate the serum, and cryopreserved at −80 °C. Prior to use in all *in vitro* and *in vivo* functional assays, the crude sera were heat-inactivated at 56 °C for 30 min to completely eliminate endogenous complement activity. Non-peptide immunized rabbit serum, subjected to the identical heat-inactivation process, was utilized as a negative control in parallel experiments.

#### 2.2.3 Determination of antibody titers by ELISA

Specific antibody titers were determined *via* enzyme-linked immunosorbent assay. Microtiter plates were coated overnight at 4 °C with 1 μg/mL polypeptide antigen diluted in carbonate-bicarbonate buffer. After PBST washing, the plates were blocked with 5% BSA at 37 °C for 1 h. Serially diluted serum samples and pre-immune negative controls were incubated at room temperature for 1 h. Subsequently, HRP-conjugated goat anti-rabbit IgG was applied for 1 h at room temperature. The reaction was developed with TMB substrate (Solarbio, Beijing, China) at 37 °C in the dark for 15 min, terminated with sulfuric acid, and the absorbance was recorded at 450 nm using a microplate spectrophotometer (Tecan, Männedorf, Switzerland). The endpoint titer was defined as the maximum serum dilution yielding an optical density greater than 2.1 times that of the negative control.

### 2.3 *In vitro* activity validation

#### 2.3.1 Bacterial strains and cell culture

*H. pylori* strain Hp5162 (Hu et al., 2015) was revived from −80°C stocks and cultured on Columbia agar (Oxoid, CM0331) supplemented with 10% sterile defibrinated sheep blood (Guangzhou Hongquan, China). The plates were incubated at 37 °C for 3–5 days under microaerophilic conditions (10% CO_2_, 5% O_2_, 85% N_2_, 95% humidity). AGS (human gastric adenocarcinoma cell line, Shanghai Cell Bank, Chinese Academy of Sciences) was cultured in RPMI 1640 medium (Gibco, Grand Island, NY, USA) containing 10% heat-inactivated fetal bovine serum (FBS, Gibco). The cells were maintained at 37 °C in a 5% CO_2_ humidified atmosphere and subcultured every 2–3 days upon reaching 80–90% confluency.

#### 2.3.2 Validation of antibody binding by using whole-cell coating of *H. pylori*

Optimal antigen and antiserum concentrations were determined *via* checkerboard titration. High-binding 96-well microtiter plates (Dynex Technologies, Chantilly, VA, USA) were coated with *H. pylori* cells (7.5 μg/mL, 100 μL/well in 0.01 M PBS, pH 7.4) for 2 h at 37 °C. After washing with PBST (PBS containing 0.05% Tween 20), the plates were blocked with 5% skim milk for 2 h at room temperature. Serially diluted rabbit antiserum and HRP-conjugated goat anti-rabbit IgG (1:5000, Abcam, Cambridge, UK) were sequentially added and incubated for 1 h at 25 °C, with PBST washes applied between the steps. The reaction was developed using TMB substrate (Solarbio, Beijing, China) in the dark for 15 min at 37 °C, terminated with 2 M H_2_SO_4_, and the absorbance was recorded at 450 nm (630 nm reference) using a microplate reader (Tecan, Mannedorf, Switzerland).

#### 2.3.3 Growth inhibition test

Blank cellulose disks (6 mm in diameter) were autoclaved and UV-sterilized. The rabbit polyclonal antiserum was sequentially filtered through 0.45 and 0.22 μm membranes, and each disk was uniformly impregnated with 25 μL of the filtrate without mechanical compression. To evaluate the bacteriostatic effect, *H. pylori* suspensions adjusted to 2 McFarland units in PBS were uniformly spread onto Columbia blood agar plates (100 μL per plate). After a brief drying period, the antiserum-impregnated disks and non-immune serum control disks were applied to the agar surface. The plates were incubated at 37 °C for 72 h under microaerophilic conditions (10% CO_2_, 5% O_2_, 85% N_2_, 95% humidity). The diameters of the zones of inhibition (ZOI) were measured using a digital caliper. All assays were performed in triplicate.

#### 2.3.4 Adhesion test

Anti-adhesion potential was evaluated *via* a modified urease-based assay (Rokka et al., 2008). *H. pylori* suspensions were pre-incubated with specific antiserum or non-immune serum (negative control) at 37 °C for 40 min. Confluent AGS cell monolayers in 96-well plates were infected with the pre-treated bacteria at a multiplicity of infection of 1:40. Following a 2 h incubation at 37 °C (5% CO_2_, 95% humidity), the wells were washed three times with PBS. Urease substrate solution (100 μL; 7 mM phosphate buffer, pH 6.8, 110 mM urea, 10 mg/L phenol red) was added to each well. After a 10–30 min reaction at room temperature, absorbance was measured at 540 nm using a microplate spectrophotometer (Tecan, Männedorf, Switzerland). Adhesion rates were calculated relative to the untreated infection control (designated as 100%), with all assays performed in triplicate.

### 2.4 *In vivo* functional evaluation

#### 2.4.1 Mice and intervention

Animal experiments were approved by the Animal Ethics Committee of the Fourth Hospital of Hebei Medical University. The overall animal experimental design is illustrated in Figure 6A. Twenty-four 5-week-old C57BL/6J mice (equal sex ratio; Liaoning Changsheng Biotechnology) were housed under specific-pathogen-free conditions. Following a standardized protocol (Lee et al., 1997), 18 mice were fasted for 12 h and gavaged with *H. pylori* (1 × 10^9^ CFU/mL, 0.2 mL) every other day for three doses. Upon verifying colonization *via* stool antigen testing, these mice were randomized into three groups (*n* = 6) for daily oral interventions: non-immune serum (*H. pylori* group), specific antiserum (Antibody group), or 20 mg/kg amoxicillin (Amoxicillin group). Six uninfected mice served as the negative control receiving vehicle treatments. All treatments were administered following a 2h fast. Upon completion, mice were euthanized *via* CO_2_ inhalation and thoracotomy. Stomachs were immediately excised and sectioned longitudinally for CFU quantification, urease activity measurement, and histomorphological analysis.

#### 2.4.2 Quantification of bacteria in mouse stomachs

Bacterial load was evaluated by homogenizing one longitudinal gastric strip from each mouse in 2 mL of sterile PBS using a tissue homogenizer. The tissue homogenates were subjected to 10-fold serial dilutions, and 100 μL aliquots of the appropriate dilutions were spread onto Columbia blood agar plates supplemented with 10% sterile defibrinated sheep blood. The plates were inverted and incubated at 37 °C under microaerophilic conditions (5% O_2_, 10% CO_2_, and 85% N_2_) with 95% relative humidity. After 4 days of incubation, *H. pylori* colonies were counted, and the colonization burden was calculated and expressed as CFU/g of stomach tissue.

#### 2.4.3 Assay for urease activity

Urease activity was assessed by mixing 0.1 mL of the gastric tissue homogenate with 0.9 mL of a urease substrate solution (7 mM phosphate buffer, pH 6.8; 110 mM urea; 10 mg/L phenol red).The mixture was incubated at 37 °C for 20 min. Following incubation, the optical absorbance of the reaction mixture was measured directly using a microplate spectrophotometer (Tecan, Männedorf, Switzerland) to quantify urease activity. To normalize these data across the different samples, the total protein concentration of the respective tissue homogenates was determined spectrophotometrically. The final relative urease activity was expressed as the optical density value per unit of total tissue protein.

#### 2.4.4 Histopathological examination

Gastric mucosal inflammation and *H. pylori* colonization were evaluated by fixing one longitudinal tissue strip from each stomach in 10% neutral-buffered formalin for 24 h. The fixed tissues were routinely processed, embedded in paraffin, and sectioned at a thickness of 4 μm. After deparaffinization and rehydration, the sections were subjected to methylene blue staining (Solarbio, Beijing, China) for 2–3 min to visualize *H. pylori* colonization, strictly following the manufacturer's protocol. The stained slides were subsequently dehydrated, cleared in xylene, mounted with neutral resin, and examined under a light microscope (Carl Zeiss, Oberkochen, Germany).

Histopathological changes in full-length longitudinal sections covering both the gastric antrum and corpus were assessed using a grading system adapted from the Updated Sydney System (Dixon et al., 1996). The following three parameters were scored independently: (i) *H. pylori* colonization density (methylene blue stain), (ii) acute inflammatory activity (neutrophilic infiltration, H&E), and (iii) chronic inflammation (mononuclear cell infiltration, H&E). Each parameter was graded on a four-point scale: 0, absent/normal; 1, mild; 2, moderate; and 3, marked/severe. Because the total experimental duration was only 4 weeks, gastric atrophy and intestinal metaplasia were uniformly recorded as score 0 for all groups and were not included as separate analytical variables.

All histological evaluations were performed independently by two experienced pathologists who were blinded to the group assignments. In cases of disagreement, a consensus score was reached by discussion. Statistical differences between groups were analyzed using the Kruskal-Wallis test followed by appropriate *post-hoc* analysis, with *P* < 0.05 considered statistically significant.

### 2.5 Statistical analysis

Statistical analyses and graph generation were performed using GraphPad Prism 9.0.0. Data are presented as the mean ± standard deviation (SD). The normality of the data distribution was evaluated using the Shapiro-Wilk test. For normally distributed data, comparisons between two groups were performed using a two-tailed paired or unpaired Student's *t*-test. Comparisons among multiple groups were evaluated using the one-way analysis of variance (ANOVA) followed by Tukey's multiple comparisons test. If the data did not pass the normality test, appropriate non-parametric tests (e.g., the Mann-Whitney U test or Kruskal-Wallis test) were applied. Statistical significance was defined as ^*^P < 0.05, ^**^P < 0.01, ^***^P < 0.001, ^****^P < 0.0001.

## 3 Results

### 3.1 Design and construction of antigen polypeptides

Existing structural evidence indicates that residues 189–244 of the BabA adhesin completely encapsulate the tripartite binding site (Moonens et al., 2016). This specific segment comprehensively includes the CL2, DL1, and DL2 loops, while also acting as the core functional region responsible for mediating specific binding to the host Le^b^ antigen. Regarding the FrpB4 nickel transporter, the fundamental rationale for selecting residues 380–395 is that this specific region simultaneously fulfills multiple stringent screening criteria. This segment is localized within the extracellular domain and concurrently exhibits strong immunogenicity and weak allergenic characteristics.

**Figure 1:**
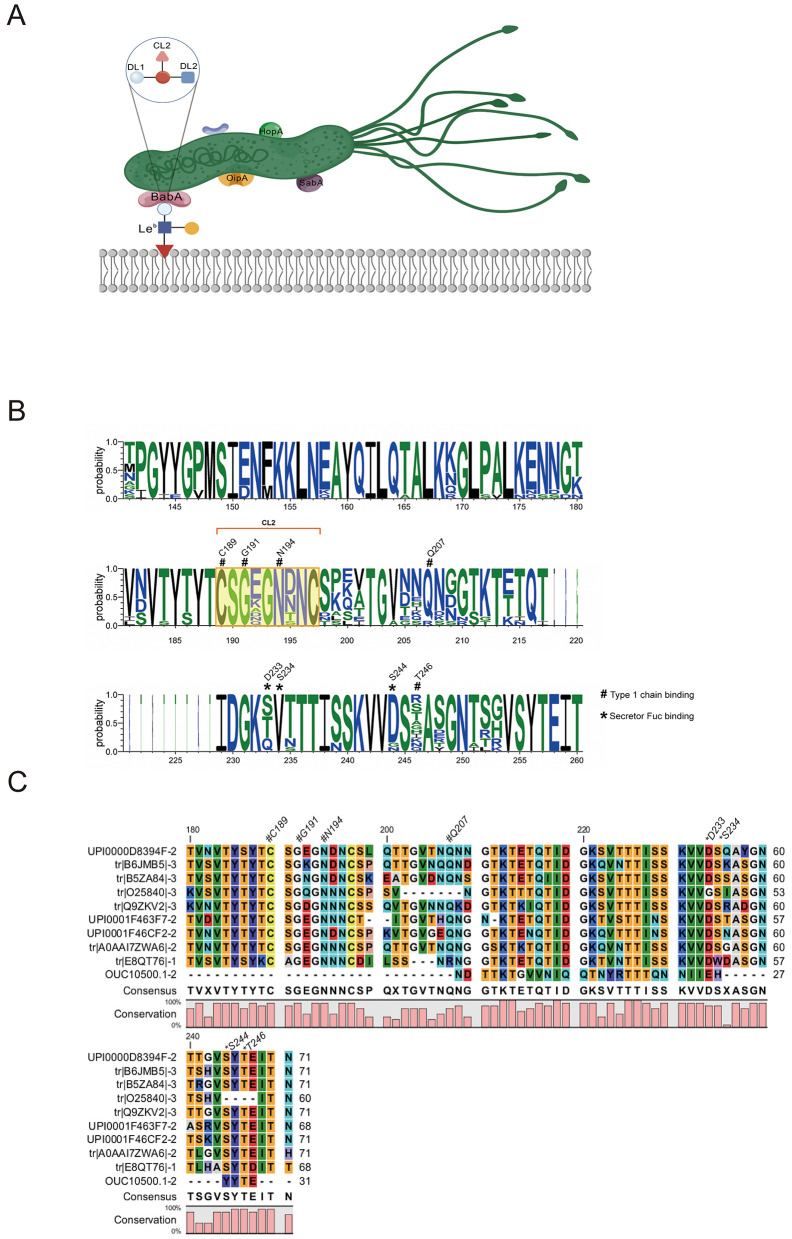
Structural basis and evolutionary conservation of the *H.pylori* BabA adhesin. **(A)** Schematic representation of BabA-mediated *H. pylori* adhesion to the Le^b^ antigen on the gastric mucosa. **(B)** Sequence conservation mapping of the BabA protein, indicating conserved structural domains. **(C)** Multiple sequence alignment of BabA amino acid sequences across 10 representative *H. pylori* strains.

**Figure 2:**
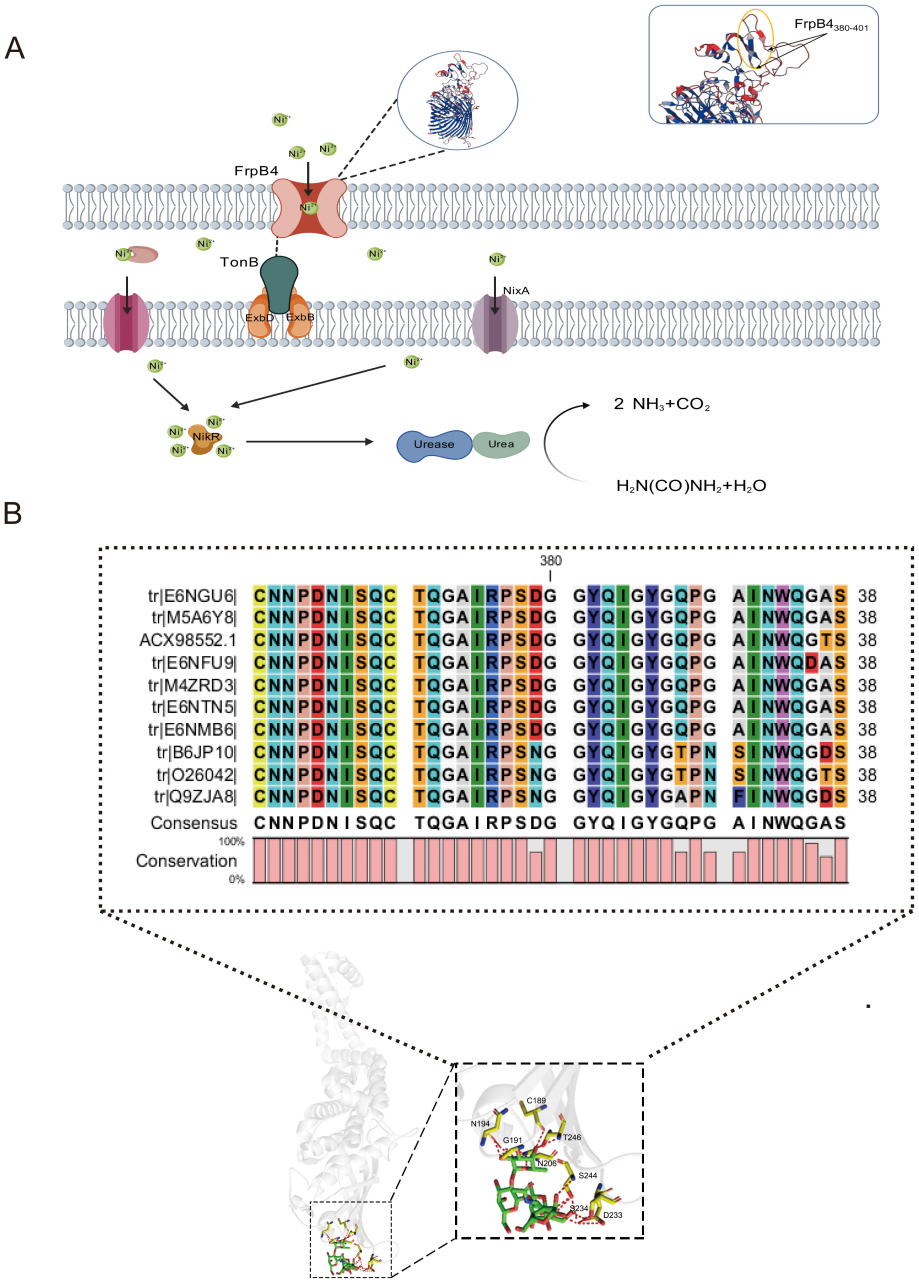
Mechanistic rationale and evolutionary conservation of targeting the FrpB4 nickel receptor. **(A)** Schematic illustration of the nickel-dependent urease activation pathway and the downstream effects of FrpB4 blockade. **(B)** Multiple sequence alignment of FrpB4 amino acid sequences across 10 representative *H. pylori* strains.

These functionally validated domains were subsequently fused to generate a chimeric antigen, establishing a dual blockade mechanism against *H. pylori* colonization (Figures 1, 2). The resulting fusion polypeptide sequence is designated as follows:

CSGEGNNNCSPQXTGVTNQNGGTKTETQTIDGKSVTTTI SSKVVDSXASGNTSGVSGGYQIGYGQPGAINWQ(BabA_189−244_: CSGEGNNNCSPQXTGVTNQNGGTKTETQTIDGKSVTTTISS KVVDSXASGNTSGVS;FrpB4_380−395_:GGYQIGYGQPGAINWQ).

### 3.2 Safety and immunogenicity of the multi-epitope antigen

**Figure 3:**
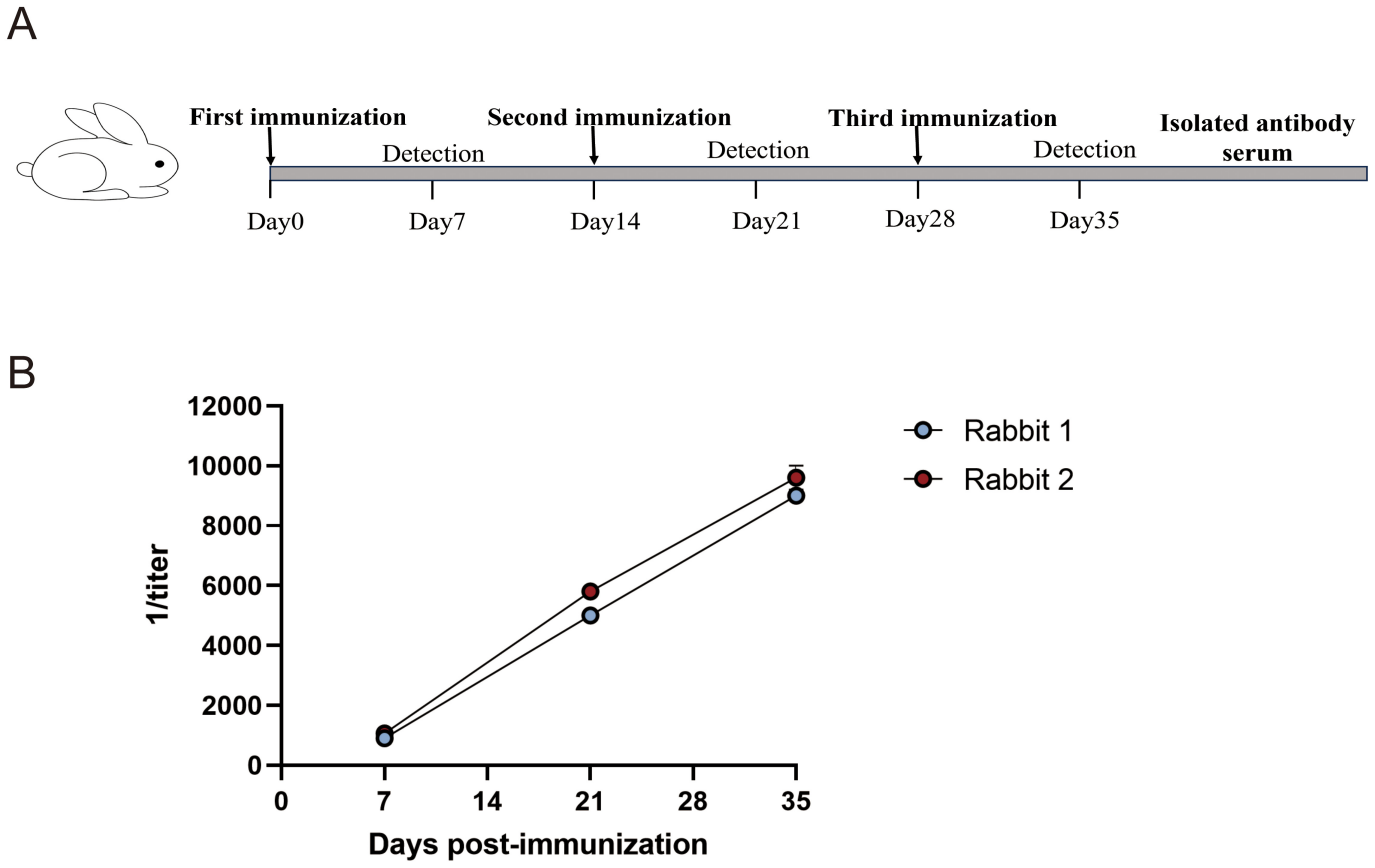
*In vivo* immunization regimen and quantitative assessment of the specific humoral immune response. **(A)** Schematic timeline of the primary and booster immunization protocol in rabbits. **(B)** Longi- tudinal specific antibody titers of two individual rabbits (Rabbit 1 and Rabbit 2) collected at 7, 21, and 35 days post-immunization. Values are expressed as the reciprocal of the specific antibody titer (1/titer).

Figure 3A outlines the experimental timeline, detailing the immunization schedule involving three doses and the subsequent blood collection points. All Japanese White rabbits maintained a stable physiological status throughout the study, presenting normal appetite and locomotor activity without any signs of systemic distress. Evaluation of the injection sites revealed a favorable safety profile characterized by the complete absence of local reactogenicity, including induration, erythema, and ulceration. Serological analysis *via* indirect ELISA confirmed the potent immunogenicity of the engineered polypeptide, demonstrating a progressive escalation in specific antibody titers after each consecutive injection. Following the final booster, the terminal antibody titer robustly exceeded 1:9,600 (Figure 3B). These quantitative findings confirm that the structural design effectively elicits a vigorous humoral immune response *in vivo*.

### 3.3 Evaluation of antibody-binding specificity and cross-reactivity

**Figure 4:**
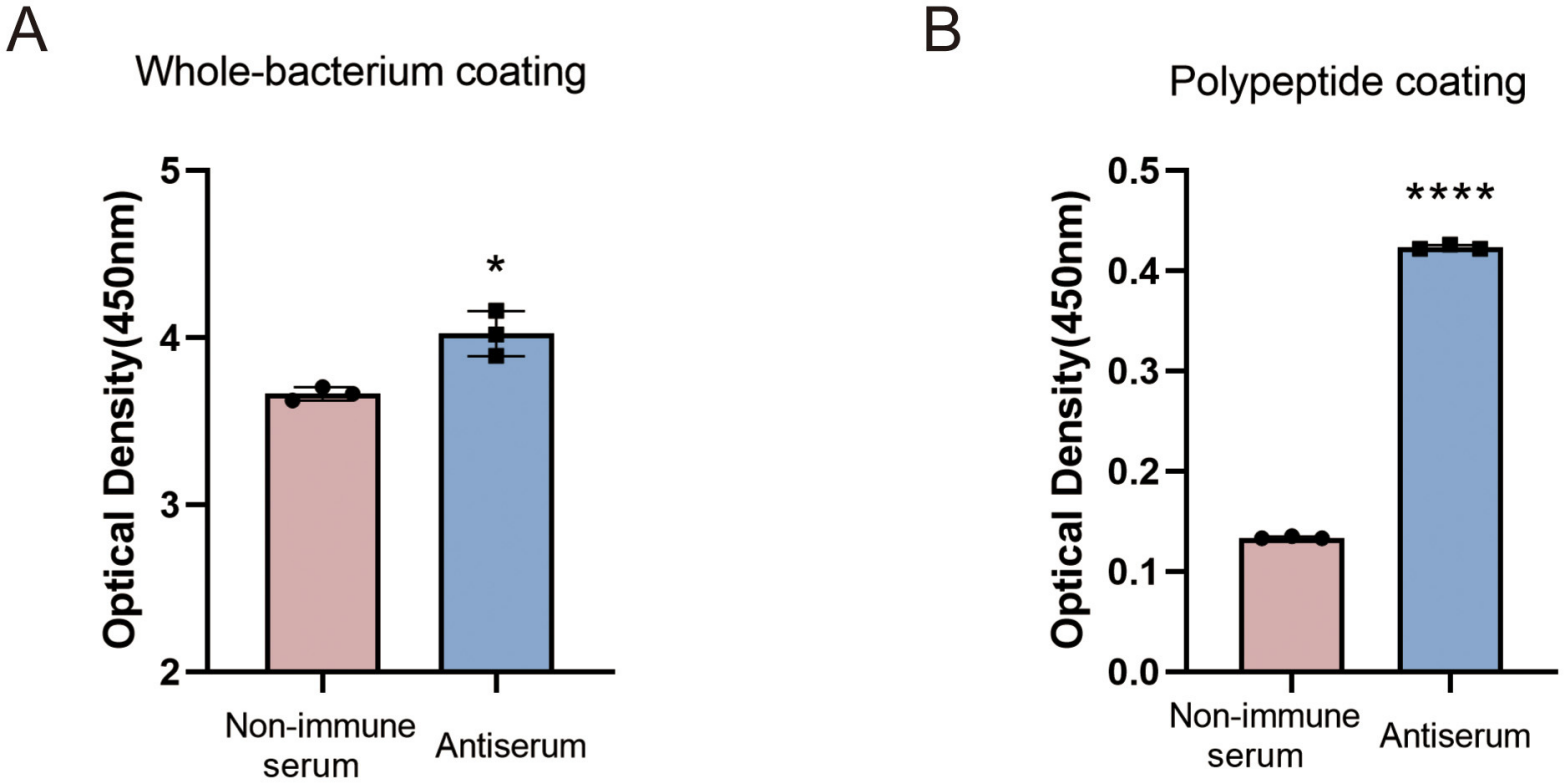
Binding specificity of the elicited antisera. **(A)** Antibody reactivity against whole *H. pylori* cells. **(B)** Antibody reactivity against the engineered multi-epitope polypeptide. Data are presented as mean ± SD. *P < 0.05, ****P < 0.0001.

We assessed the recognition capacity of the elicited antisera by evaluating the binding reactivity of post-immunization serum against intact *H. pylori* cells and the corresponding synthetic polypeptide *via* ELISA. The post-immunization serum demonstrated significantly enhanced binding to native *H. pylori* cells compared to the pre-immune control (Figure 4A). This provides direct evidence that the antibodies elicited by the polypeptide can effectively recognize the intact bacterium. Furthermore, the antisera exhibited strong specific binding toward the purified recombinant polypeptide alongside minimal background reactivity (*P* < 0.0001; Figure 4B). These results collectively indicate that the elicited antibodies demonstrate surface reactivity against the intact bacterium. However, constrained by the complex composition of polyclonal antibodies, this assay is incapable of distinguishing the specific binding interactions of individual target proteins.

### 3.4 *In vitro* antibacterial activity of antiserum-induced antibodies

**Figure 5:**
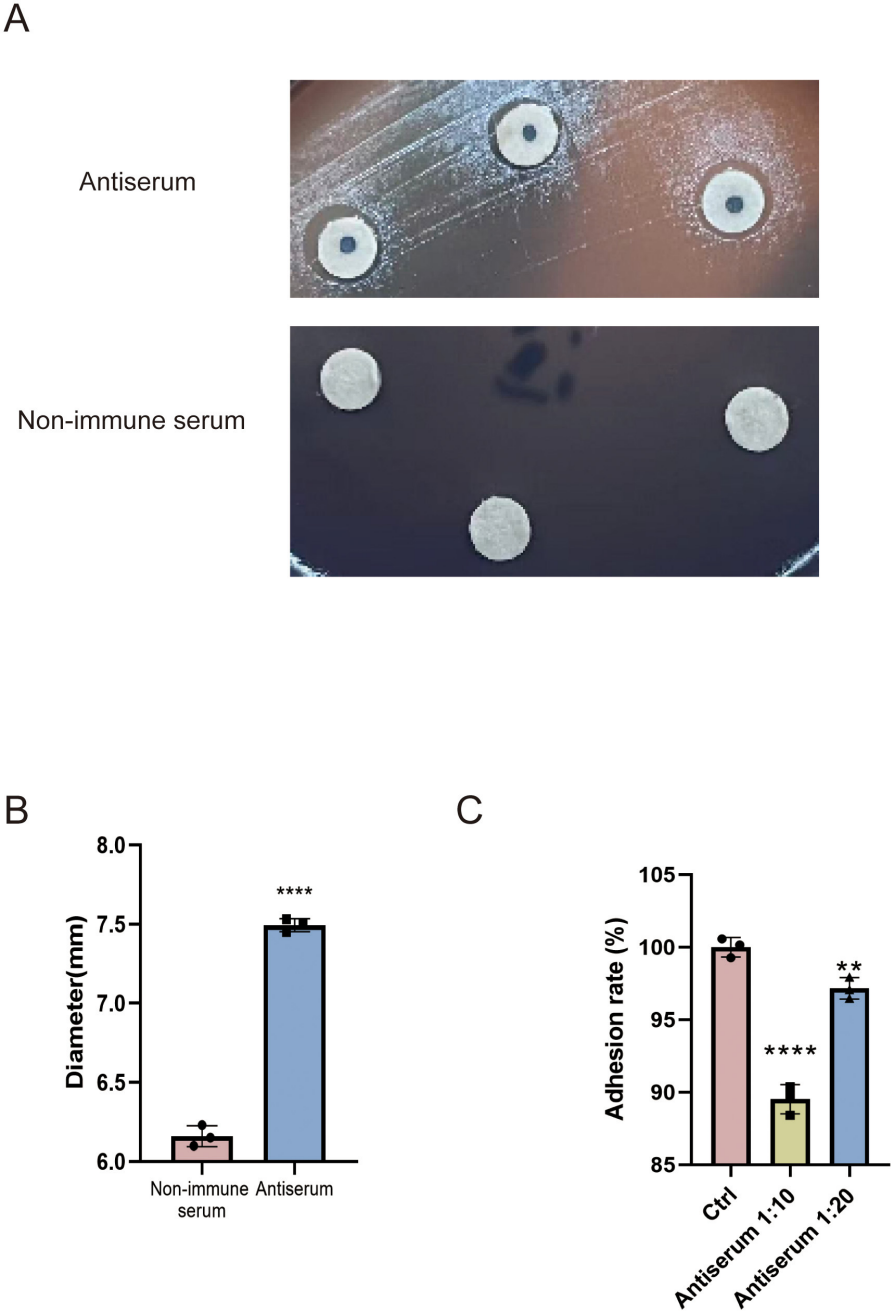
*In vitro* functional validation of the dual-target neutralizing antisera. **(A)** Representative images of the disk diffusion assay against *H. pylori* cultures. **(B)** Quantitative analysis of the inhibition zone diameters. **(C)** Quantitative assessment of *H. pylori* adhesion to AGS cells following antiserum pre-treatment. Data are presented as mean ± SD. *P < 0.01, ****P < 0.0001.

A disk diffusion assay assessed the inhibitory capacity of the elicited antibodies against *H. pylori*. Amoxicillin served as a positive control and yielded prominent inhibitory zones, confirming the sensitivity of the experimental system. While disks impregnated with non-immune serum showed no detectable suppression of bacterial growth, those treated with specific antibodies produced clear inhibitory zones (Figure 5A). The zone diameters in the antibody-treated group were markedly larger than those in the negative control (Figure 5B). These findings demonstrate that the multiepitope polypeptide induces antibodies capable of effectively restricting *H. pylori* proliferation *in vitro*.

### 3.5 Inhibition of *H. pylori* adhesion

The induced polyclonal antibodies partially but significantly reduced *H. pylori* attachment to AGS cells *in vitro*. A clear concentration-dependent suppression of pathogen adhesion was observed during the cellular co-culture. Application of the antibodies at a 1:20 dilution slightly reduced the bacterial adhesion rate to 97.16% relative to the untreated control. A higher concentration (1:10 dilution) further decreased the relative adhesion rate to 89.52%(*P* < 0.05, Figure 5C).

### 3.6 Therapeutic efficacy of antiserum on *H. pylori* gastric colonization and urease activity

**Figure 6:**
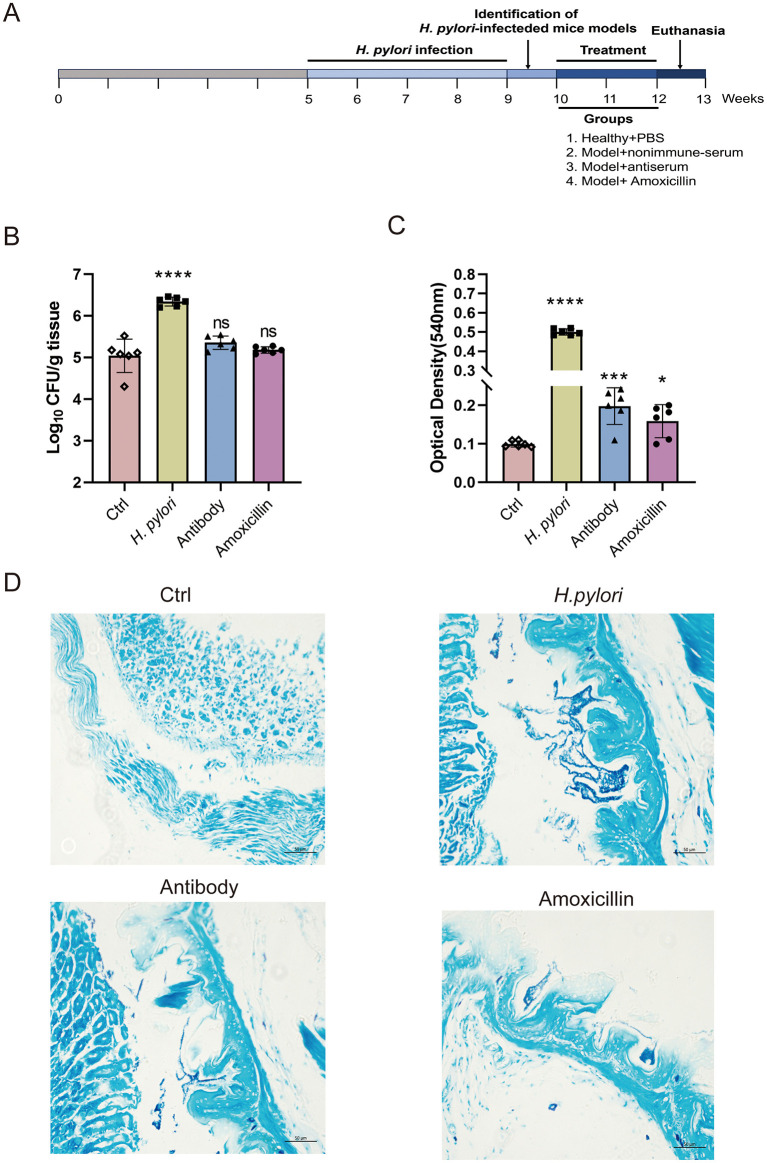
*In vivo* evaluation of the dual-target antisera in a murine *H. pylori* infection model. **(A)** Schematic timeline encompassing *H. pylori* colonization and subsequent oral antiserum administration. **(B)** Quantification of the gastric bacterial load from homogenized gastric tissues across experimental groups. **(C)** Quantitative analysis of urease activity in gastric tissues measured by optical density at 540 nm. **(D)** Representative histological images of the gastric mucosa stained with Methylene Blue for the visualization of *H. pylori* colonization. Data are presented as mean ± SD. ns: not significant, *P < 0.05, ****P < 0.001, ****P < 0.0001.

Oral administration of the targeted antiserum significantly restricted *H. pylori* gastric colonization and suppressed mucosal urease activity *in vivo*. Quantitative analysis revealed a substantial bacterial burden in the untreated infected group compared with the uninfected control (*P* < 0.0001). Conversely, intervention with the specific antiserum caused a pronounced reduction in gastric bacterial density (Figure 6B). Parallel evaluations of mucosal urease activity aligned strictly with the quantitative culture outcomes. The elevated urease levels characterizing the untreated cohort were significantly mitigated following the oral delivery of the specific antiserum (Figure 6C).

### 3.7 Histological evaluation of gastric colonization by *H. pylori*

Methylene blue staining and hematoxylin and eosin (H&E) staining provided *in situ* histological confirmation of the reduced *H. pylori* colonization and mucosal inflammation following oral immunotherapy. Gastric tissues from the untreated infected cohort exhibited a high density of bacteria adhering directly to the epithelial surface, accompanied by marked inflammatory infiltration. Conversely, administration of the targeted polyclonal antibodies substantially decreased the abundance of mucosa-adherent bacteria (Figure 6D). Pathological evaluation using the Updated Sydney System revealed that the antibody-treated group showed significantly lower scores in both acute neutrophilic activity and chronic mononuclear inflammation compared to the untreated *H. pylori* infection group (*P* < 0.05, Supplementary Figure S1). These histopathological scorings firmly align with the quantitative culture outcomes.

## 4 Discussion

This study evaluated an immunotherapeutic strategy simultaneously targeting the BabA adherence domain and the metal metabolism channel FrpB4. Experimental results demonstrated that the induced specific antibodies exerted anti-adhesive and bacteriostatic effects *in vitro*, and reduced the colonization load of *H. pylori* in the mouse stomach. Given the growing threat of antimicrobial resistance (Li et al., 2023; Gravina et al., 2018; Fallone et al., 2019; Yu et al., 2024), non-antibiotic strategies targeting well-defined virulence factors merit systematic research. Unlike the broad and heterogeneous immune responses induced by whole-cell immunogens (Marnila et al., 2003; Shin et al., 2002; Hu et al., 2015), structure-guided subunit antigen design focuses antibody responses on highly conserved pathogen targets. This approach can minimize off-target reactivity and restrict bacterial immune evasion (Rappuoli et al., 2016; Serruto et al., 2012; Meza et al., 2017; Keshri et al., 2023).

The structural features of receptor-binding domains lay the groundwork for neutralizing antibody design. Although *H. pylori* employs phase variation of outer membrane proteins to adapt to harsh host environments (Xu et al., 2020; Moonens et al., 2016), the receptor-binding cleft of the BabA adhesin must maintain strict conformational stability to mediate bacterial adhesion to host cells (Keilberg and Ottemann, 2016; Oleastro and Ménard, 2013; Ilver et al., 1998). However, from the perspective of pathogenic biology, *H. pylori* possesses a functionally highly redundant family of outer membrane proteins that collectively mediate host cell attachment, including SabA, AlpA/B, and HpaA (Aspholm et al., 2006). Therefore, blocking a single BabA molecule is theoretically insufficient to completely abrogate bacterial adhesion due to the compensatory expression of other adhesion pathways. The partial adhesion inhibition detected in our *in vitro* assays indicates that antibodies induced by the synthetic antigen exert specific steric hindrance at the host pathogen interface. Achieving robust adhesion blockade in the future will require a multi-target synergistic intervention strategy. These *in vitro* functional findings are consistent with the reduced gastric colonization observed *in vivo*, which was quantified via viable bacterial counting and further validated by histological evaluations and urease activity assays (Rokka et al., 2008; Mobley et al., 1991; Ansari and Yamaoka, 2017).

When evaluating the *in vitro* bacteriostatic activity of macromolecular antibodies, the agar diffusion assay serves as a robust analytical tool offering rapid, direct, and macroscopic visualization of bacteriostatic efficacy. Nevertheless, the physical limitations of this system must be taken into account. Full-length IgG molecules have a high molecular weight and diffuse slowly in dense agar matrices. Thus, caution is required when quantitatively comparing their diffusion characteristics with those of small-molecule antibiotics (Nguyen et al., 2019; Hurley and Theil, 2011). Furthermore, biophysical studies have established that *H. pylori* secretes urease to hydrolyze urea and generate ammonia, triggering a gel to sol transition of the gastric mucin from a solid-like elastic gel to a liquid-like viscous solution (Celli et al., 2009). This local liquefaction significantly reduces the physical resistance for antibodies *in vivo*. Therefore, the disk diffusion model represents a conservative physical barrier scenario. The observed formation of distinct inhibition zones confirms localized growth suppression. We speculate that the observed growth arrest of *H. pylori* may be largely attributed to steric hindrance of the FrpB4 protein, though this mechanism remains unconfirmed. *H. pylori* relies critically on metalloenzymes such as urease and hydrogenase for survival (Mobley et al., 1991; Ansari and Yamaoka, 2017, 2019). Antibodies targeting the extracellular loops of FrpB4 (De Reuse et al., 2013; Schauer et al., 2007; Wolfram et al., 2006) could potentially disrupt the uptake of vital nutrients. Nevertheless, our current work does not provide direct microscopic proof of intracellular metal ion depletion or quantitative measurements of nickel import, so this proposed interference in nutrient acquisition is only a tentative hypothesis that demands further dedicated functional experiments to verify.

*In vivo* results showed that oral delivery of the specific antiserum significantly reduced the viable bacterial burden by 0.8 log_10_ CFU/g in the stomach of C57BL/6J mice (Lee et al., 1997; Arnold et al., 2011; Burns et al., 2017). Although the number of viable bacteria decreased markedly, residual urease activity in gastric tissue remained above the baseline level of uninfected mice. This temporal discrepancy is consistent with fundamental pharmacokinetic and enzymatic properties. Specific antibodies can rapidly suppress bacterial replication through steric hindrance and nutrient deprivation. In contrast, free urease metalloenzymes secreted into the gastric mucin layer have an independent degradation half-life, and the complete clearance of these large enzyme complexes requires an extended duration. This finding explains the biological rationale behind clinical guidelines recommending that urea breath tests be performed 4–6 weeks after *H. pylori* eradication (Malfertheiner et al., 2022), and accounts for the inherent time lag between bacterial clearance and the degradation of residual metabolic markers.

The translational potential of the present study must be interpreted in light of several physiological and methodological limitations. Oral delivery of unencapsulated antibodies inherently faces extreme proteolytic challenges in the stomach. Although pepsin cleaves IgG into F(ab′)_2_ fragments that retain bivalent antigen-binding capacity, and the urease-mediated pH elevation provides a temporary micro-neutralizing environment (Jasion and Burnett, 2015), the lack of *in situ* gastric stability monitoring and intragastric antibody recovery data in this murine model remains a notable constraint. While previous clinical trials demonstrated the macroscopic clearance effect of oral anti *H. pylori* antibodies (Hu et al., 2015; Guo et al., 2021), acid resistant encapsulation technologies, such as chitosan matrices (Amaral et al., 2025), are essential for reliable clinical translation. Beyond delivery challenges, the current experimental design requires further mechanistic depth. The whole-cell ELISA confirmed broad-spectrum surface reactivity but could not distinguish single-protein binding specificities. Direct functional assays, including urease maturation and FrpB4-specific knockout evaluations utilizing monoclonal antibodies, are therefore essential to definitively confirm the specific ion channel blockade mechanism. In terms of *in vivo* evaluations, the localized mucosal immune microenvironment remains uncharacterized due to the absence of cytokine and immune cell profiling. Overall, this antibody candidate provides a feasible foundation for anti-*H. pylori* oral therapy. To advance this structural design toward clinical translation, further rigorous investigations are warranted: *in vitro* binding tests using human gastric and intestinal cell lines to assess off-target cross-reactivity, and functional validation against a geographically diverse panel of *H. pylori* strains to confirm its broad-spectrum potency.

## 5 Conclusion

This study demonstrates the potential of a structure-based strategy targeting both BabA-mediated adhesion and FrpB4-dependent nickel acquisition. The elicited multi-epitope antibodies exhibited clear neutralizing activity *in vitro* and substantially reduced gastric colonization *in vivo*, supporting the concept that steric blockade of key outer membrane nodes can disrupt early pathogenesis. Although the oral antiserum delivery model faces translational limitations due to gastric degradation, the confirmed antibody affinity and *in vivo* clearance capacity provide a foundation for further development. Future translation will require integrating these antigenic targets with engineered antibody formats, utilizing gastric targeted delivery systems, and conducting functional evaluations against globally diverse *H. pylori* strains to confirm broad spectrum efficacy. If successful, this approach may offer a valuable non-antibiotic option against multidrug resistant *H. pylori*.

## Data Availability

The original contributions presented in the study are included in the article/Supplementary material, further inquiries can be directed to the corresponding authors.
