# Supplementary material for: Therapeutic suppression of Helicobacter pylori infection in C57BL/6J mice using specific polyclonal antibodies targeting BabA and FrpB4
Source: Front Microbiol. 2026 Jul 31;17:1867770. doi: 10.3389/fmicb.2026.1867770 (PMC13473165; doi:10.3389/fmicb.2026.1867770)
Supplement: Supplementary file 1 [file Data_Sheet_1.docx]

**Supplementary Material 1**

**Table S1. List of *Helicobacter pylori* strains and corresponding BabA accession numbers used for sequence homology analysis.**

| ***H. pylori* Strain** | **Accession Number** | **Database Source** |
| --- | --- | --- |
| Strain HPAG1 | UPI0000D8394F | UniProt |
| Strain P12 | B6JMB5 | UniProt |
| Strain G27 | B5ZA84 | UniProt |
| Strain 26695 | O25840 | UniProt |
| Strain J99 | Q9ZKV2 | UniProt |
| Strain F30 | UPI0001F463F7 | UniProt |
| Strain F57 | UPI0001F46CF2 | UniProt |
| Strain 51 | A0AAI7ZWA6 | UniProt |
| Strain SouthAfrica7 | E8QT76 | UniProt |
| *Helicobacter pylori* SS1 | OUC10500.1 | UniProt |

**Table S2. List of *Helicobacter pylori* strains and corresponding FrpB4 accession numbers used for sequence homology analysis.**

| ***H. pylori* Strain** | **Accession Number** | **Database Source** |
| --- | --- | --- |
| strain F30 | E6NGU6 | UniProt |
| strain OK113 | M5A6Y8 | UniProt |
| strain 51 | ACX98552.1 | UniProt |
| strain F16 | E6NFU9 | UniProt |
| strain OK310 | M4ZRD3 | UniProt |
| strain F57 | E6NTN5 | UniProt |
| strain F32 | E6NMB6 | UniProt |
| strain P12 | B6JP10 | UniProt |
| strain 26695 | O26042 | UniProt |
| strain J99 | Q9ZJA8 | UniProt |

Supplementary Figure S1

**Supplementary Figure S1. Mass spectrometry analysis of the synthesized multi-epitope polypeptide.**

**Supplementary Figure S2**

**Supplementary Figure S2.** **Histopathological scores of gastric lesions.** (A) *H. pylori* colonization density score; (B) Acute neutrophilic inflammation score; (C) Chronic inflammatory infiltration score. Each dot represents one mouse, and dashed lines indicate group medians. Ctrl vs Hp comparisons were analyzed by Mann-Whitney U test. Three-group comparisons (Hp, Antibody, Amo) were performed via Kruskal-Wallis test with Dunn’s post-hoc test. Asterisks denote significant differences relative to the Hp group: **P* < 0.05, ***P* < 0.01.
