# Supplementary material for: Therapeutic suppression of Helicobacter pylori infection in C57BL/6J mice using specific polyclonal antibodies targeting BabA and FrpB4
Source: Front Microbiol. 2026 Jul 31;17:1867770. doi: 10.3389/fmicb.2026.1867770 (PMC13473165; doi:10.3389/fmicb.2026.1867770)
Supplement: Supplementary file 2 [file Data_Sheet_2.docx]

**Supplementary Table S3. Summary of Phylogeographic Origins and Pathogenic Features for 14 Globally Diverse *Helicobacter pylori* Strains.**

| Strain Name | Accession/Assembly No. | Country/Region of Isolation | Disease Source | *cag*-PAI & *VacA* Genotype |
| --- | --- | --- | --- | --- |
| 26695 | GCF_000307795.1/ CP003904.1 | United Kingdom | Chronic gastritis | Intact, *cagA* positive (s1b/m1) |
| J99 | GCF_000008785.1 / AE001439.1 | United States | Duodenal ulcer | Intact, *cagA* positive (s1b/m1) |
| HPAG1 | GCF_000013245.1 / CP000241.1 | Sweden (Europe) | Chronic atrophic gastritis | Intact, *cagA* positive (s1b /m1) |
| P12 | GCF_000021165.1 / CP001217.1 | Germany | Peptic ulcer | Intact, *cagA* positive (s1a/m1) |
| G27 | GCF_000021285.1 / CP001173.1 | Italy | Duodenal ulcer | Intact, *cagA* positive (s1a/m2) |
| F30 | GCF_000270025.1 / AP011941.1 | Fukui, Japan (East Asia) | Duodenal ulcer | Defective *cag*-PAI, *cagA* positive (s1b/m1) |
| F57 | GCF_000270065.1 / AP011945.1 | Fukui, Japan (East Asia) | Gastric cancer | Defective cag-PAI , *cagA* positive (s1b/m1) |
| F16 | GCF_000270005.1 / AP011940.1 | Fukui, Japan (East Asia) | Gastritis | Defective *cag*-PAI , *cagA* positive (s1c/m1) |
| F32 | GCF_000270045.1 / AP011943.1 | Fukui, Japan (East Asia) | Gastric cancer | Defective *cag*-PAI , *cagA* positive (s2/m1) |
| 51 | GCF_000011725.1 / CP000012.1 | South Korea | Duodenal ulcer | Intact, *cagA* positive (s1/m1) |
| SA7 (SouthAfrica7) | GCA_000270025.1 / CP002336.1 | South Africa | Dyspepsia / Not specified | Intact, *cagA* positive (s1a/m1) |
| SS1 | GCF_002005525.1 / CP009259.1 | Australia (mouse-adapted) | Chronic gastritis | Defective *cag*-PAI (non-functional T4SS), *cagA* positive (s2/m2) |
| OK113 | GCF_000348865.1 / AP012600.1 | Okinawa, Japan (East Asia) | Gastric cancer | Intact, *cagA* positive (s1c/m1) |
| OK310 | GCF_000348885.1 / AP012601.1 | Okinawa, Japan (East Asia) | Duodenal ulcer | Intact, *cagA* positive (s1c/m1) |
